# Maternal Pravastatin Prevents Altered Fetal Brain Development in a Preeclamptic CD-1 Mouse Model

**DOI:** 10.1371/journal.pone.0100873

**Published:** 2014-06-25

**Authors:** Alissa R. Carver, Maria Andrikopoulou, Jun Lei, Esther Tamayo, Phyllis Gamble, Zhipeng Hou, Jiangyang Zhang, Susumu Mori, George R. Saade, Maged M. Costantine, Irina Burd

**Affiliations:** 1 Division of Maternal Fetal Medicine, Department of Obstetrics and Gynecology, University of Texas Medical Branch, Galveston, Texas, United States of America; 2 Integrated Research Center for Fetal Medicine, Division of Maternal Fetal Medicine, Department of Gynecology and Obstetrics, Johns Hopkins University, Baltimore, Maryland, United States of America; 3 The Russell H. Morgan Department of Radiology and Radiological Science, Johns Hopkins University, Baltimore, Maryland, United States of America; Florey Institute of Neuroscience and Mental Health, The University of Melbourne, Australia

## Abstract

**Objective:**

Using an animal model, we have previously shown that preeclampsia results in long-term adverse neuromotor outcomes in the offspring, and this phenotype was prevented by antenatal treatment with pravastatin. This study aims to localize the altered neuromotor programming in this animal model and to evaluate the role of pravastatin in its prevention.

**Materials and Methods:**

For the preeclampsia model, pregnant CD-1 mice were randomly allocated to injection of adenovirus carrying sFlt-1 or its control virus carrying mFc into the tail vein. Thereafter they received pravastatin (sFlt-1-pra “experimental group”) or water (sFlt-1 “positive control”) until weaning. The mFc group (“negative control”) received water. Offspring at 6 months of age were sacrificed, and whole brains underwent magnetic resonance imaging (MRI). MRIs were performed using an 11.7 Tesla vertical bore MRI scanner. T2 weighted images were acquired to evaluate the volumes of 28 regions of interest, including areas involved in adaptation and motor, spatial and sensory function. Cytochemistry and cell quantification was performed using neuron-specific Nissl stain. One-way ANOVA with multiple comparison testing was used for statistical analysis.

**Results:**

Compared with control offspring, male sFlt-1 offspring have decreased volumes in the fimbria, periaquaductal gray, stria medullaris, and ventricles and increased volumes in the lateral globus pallidus and neocortex; however, female sFlt-1 offspring showed increased volumes in the ventricles, stria medullaris, and fasciculus retroflexus and decreased volumes in the inferior colliculus, thalamus, and lateral globus pallidus. Neuronal quantification via Nissl staining exhibited decreased cell counts in sFlt-1 offspring neocortex, more pronounced in males. Prenatal pravastatin treatment prevented these changes.

**Conclusion:**

Preeclampsia alters brain development in sex-specific patterns, and prenatal pravastatin therapy prevents altered neuroanatomic programming in this animal model.

## Introduction

Preeclampsia complicates 5–8% of pregnancies and causes significant maternal and neonatal morbidity and mortality [1]. Currently, the only effective therapy is delivery of the pregnancy, which can lead to prematurity and its associated risks. Though the ultimate etiology of preeclampsia remains elusive, research has focused on abnormal vascular adaptation during placental development. Specifically, abnormal trophoblast invasion and spiral artery remodeling lead to a hypoxic placenta, release of vasoactive factors and cytokines into the maternal circulation, and endothelial dysfunction [2]. This produces a syndrome of maternal hypertension, proteinuria, and other end-organ damage.

To investigate preeclampsia, we and others have used an animal model with a preeclampsia-like phenotype induced by over-expression of the anti-angiogenic soluble FMS like tyrosine kinase (sFlt-1) using an adenovirus vector. This model is based on evidence that women with pre-eclampsia have elevated serum sFlt-1, decreased vascular endothelial growth factor (VEGF) and placental growth factor (PlGF), and impaired angiogenesis [3]–[4]. Overexpressing sFlt-1 in the murine model leads to hypertension, altered vascular and endothelial responses, and other manifestations of preeclampsia [5]–[6]. In addition, we showed that pups born to “preeclamptic” dams are more susceptible to hypoxic ischemic injury, have altered candidate gene expression in the brain, and as adults perform worse on neuromotor assays testing vestibular function, balance, and locomotor coordination compared with pups born to control mothers, all in a gender-specific manner [7]–[8]. These findings are in accordance with various epidemiologic studies showing altered neuromotor fetal programming in infants born to preeclamptic mothers [9]–[14].

Due to the similarities between preeclampsia and adult cardiovascular disease, we and others have shown, using animal models of preeclampsia, that antenatal treatment with pravastatin leads to improved blood pressures, vascular reactivity and pup growth [15]–[17]. Further, treatment with pravastatin increased VEGF and PlGF while decreasing sFlt-1 [18]–[19]. These findings have occurred with no increase in pup resorption, deformation, or changes in maternal cholesterol levels [16]–[17]. Additionally, we demonstrated that prenatal treatment of these mice with pravastatin prevented fetal programming of abnormal neuromotor function and gene expression in the offspring. Specifically it restored performance on assays testing vestibular function, balance, and locomotor coordination as well as expression of genes involved in neuronal migration, myelination, apoptosis and oxidative stress to levels similar to control (unpublished data) [8]. The objectives of this study were to localize regions of the brain that are associated with preeclampsia's fetal programming of long-term neuromotor abnormalities and to determine the effect of maternal therapy with pravastatin in these regions.

## Materials and Methods

### Ethics Statement

This study was carried out in strict accordance with the recommendations in the Guide for the Care and Use of Laboratory Animals of the National Institutes of Health. All procedures involving animals were approved by the Institutional Animal Care and Use Committee (IACUC) at the University of Texas Medical Branch, Galveston, Texas (Protocol #0411077) and Johns Hopkins University, Baltimore, Maryland (Protocol #M011M420). All efforts were made to minimize suffering.

### Animals

Timed pregnant CD-1 mouse dams (Charles Rivers Laboratories, Wilmington, MA) were housed in groups of no more than 6 in a temperature- and humidity-controlled room in 12 hour light-dark cycle with access to regular chow and water *ad libitum*. Certified personnel and veterinary staff provided regular maintenance and animal care according to our IACUC guidelines.

### Experimental Protocol

The preparation of adenovirus carrying sFlt-1 and the murine immunoglobulin G2Fc (mFc) fragments, as well as the generation and validation of the preeclampsia-like model, have been described in detail elsewhere [4]–[6]. In brief, we used a replication-deficient recombinant adenovirus vector that leads, after a single injection, to hepatocyte transduction, which allows sustained in vivo transgene expression and secretion into the systemic circulation.

At day 8 of gestation, pregnant CD-1 mice were randomly allocated to tail vein injection with either adenovirus carrying sFlt-1 (10^9^ plaque-forming units in 100 µL) or adenovirus carrying equal concentration of the murine immunoglobulin G2Fc (mFc group; control). The sFlt-1 dams were then allocated to receive either water (sFlt-1 group) or pravastatin (sFlt-1-pra). Pravastatin (Sigma-Aldrich, St Louis, MO) was dissolved in water at a concentration that gives a final dose of 5 mg/kg/d based on the daily water consumption of pregnant mice, which has been previously determined [15]. The mFc group received water. This protocol resulted in the following groups: mFc, sFlt-1, and sFlt-1-pra. An mFc-pravastatin group was not used as previous studies in our laboratory showed no adverse outcomes for this group [15]–[16]. Mice were allowed to deliver on days 19–21 of gestation, and maternal treatment allocation was continued through weaning. Offspring were culled, housed in sibling groups, and fed a standard laboratory diet.

At 6 months of age, male [(mFc (n = 9), sFlt-1 (n = 3), and sFlt-1-pra (n = 6)] and female [(mFc (n = 6), sFlt-1 (n = 3), and sFlt-1-pra (n = 10)] offspring were anesthetized using 1% ketamine/xylazine by intraperitoneal injection. Mice were perfused with 4% ice-cold paraformaldehyde after transcardiac perfusion of sterile normal saline. Whole brains were dissected and post-fixed in 4% paraformaldehyde at room temperature for a minimum of 1 week to ensure proper fixation. Prior to imaging, brain samples were then transferred into phosphate-buffered saline (PBS) for 3 days to wash out paraformaldehyde.

### Brain Imaging

Whole brains underwent magnetic resonance imaging (MRI). We performed ex vivo MRI studies on a vertical bore 11.7 Tesla magnetic resonance scanner (Bruker Biospin, Billerica, MA) using a 15-mm diameter volume coil as the radiofrequency transmitter and receiver. Three-dimensional T2 weighted images of mouse brains were acquired using a fast spin echo sequence with the following parameters: echo time (TE)/repetition time (TR)  = 40/2000 ms, resolution  = 0.08 mm×0.08 mm×0.08 mm, echo train length  = 8, number of average  = 2. The imaging resolution and contrast were sufficient for automatic volumetric characterization of the mouse brains and 28 substructures of interest (Table 1). The detailed image analysis was described in our previous study [20]. Briefly, two blinded examiners manually processed the magnetic resonance (MR) images. Using an existing mouse brain atlas, which is based on T2-weighted images and structural segmentation according to Paxinos' atlas [21], the atlas and its corresponding structural segmentations were transformed together with those of the individual subject images. Images were analyzed using DiffeoMap software (www.mristudio.org) to allow quantitative measurement of volume changes between the template and subject images.

**Table 1:** Regions of Interest Imaged Via MRI in the Brains of 6-Month Old Male and Female Offspring.

Regions of Interest	
Accumbens Nucleus	Inferior Colliculus
Amygdala	Internal Capsule
Anterior Commissure	Lateral Globus Pallidus
Caudate Putamen	Neocortex
Cerebellum	Olivary Pretectal Nucleus
Cingulum	Periaqueductal Gray
Claustrum	Piriform Cortex
Endopiriform Nucleus	Septum Pellucidum
External Capsule	Stria Medullaris
Fasciculus Retroflexus	Submammillothalamic Nucleus
Fimbria	Stria Terminalis
Fornix	Superior Colliculus
Hippocampus	Thalamus
Hypothalamus	Ventricles

### Cytochemistry

As previous work in this model has shown alteration in gene expression of a neuronal migration protein (unpublished data), we selected Nissl stain. Following MRI acquisition, whole brains were washed in 1X PBS for 30 min five times. The samples were processed for histochemical staining by immersing in 30% sucrose until saturation and cryosectioned at 20 µm thickness. Nissl staining for endoplasmic reticulum granular bodies was performed by using Cresyl violet. Briefly, sections were incubated with a 0.5% solution of Cresyl echt violet (Chroma-Gesellschaft, Roboz, Inc., Gaithersburg, MD) in sodium acetate buffer for 5 min followed by a brief rinse in tap water. The stain was differentiated in 1% glacial acetic acid in 95% ethanol until white matter tracts were visible from gray matter. After dehydration with gradient ethanol, the tissue was cleared in Hemo-De and mounted with distyrene plasticizer and xylene (Fluka Chemical, Sigma-Aldrich, St. Louis, MO). The neurons were identified by dark blue-positive staining. All photographs used for quantification were taken with Zeiss AxioPlan 2 Microscope System (Jena, Germany) attached to a Canon EOS Rebel Camera (Tokyo, Japan) through a 40 objective lens. Cell count was performed by Image J 1.37V (National Institute of Health, Bethesda, MD) on randomly chosen 7-8 fields in frontal cortex per animal. The experiments were performed in triplicate for all treatment groups.

### Statistical Analysis

Analysis was performed using Prism 5 (GraphPad Software Inc., La Jolla, CA). For tissue volume comparison, Shapiro-Wilk test was used to assess for normality and one-way ANOVA with Newman-Keuls multiple comparison analysis was performed. Two-way ANOVA was performed on cell count data with post-hoc Bonferroni test. Data are reported as mean ± SEM. A two-tailed p<0.05 was considered statistically significant.

## Results

**Figure 1:**
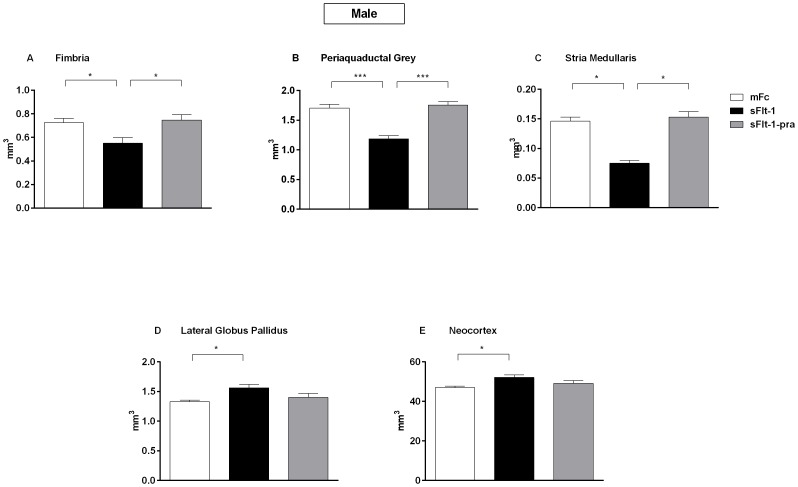
Altered Brain Volumes in 6 Month Old Male Offspring Brain. Regions of interest were analyzed by 1-way ANOVA in 6 month old male mice offspring born to pregnant CD1 mice injected either with an adenovirus carrying sFlt-1 or adenovirus carrying mFc fragment. Mice injected with sFlt-1 were treated with either pravastatin or water to result in three groups of offspring animals: mFc (n = 9), sFlt-1 (n = 3), and sFlt-1-pra (n = 6). Data are reported as mean ± SEM. The sFlt-1 group offspring showed decreased volumes at the fimbria (A; p = 0.04), periaqueductal grey (B; p<0.0001), and stria medullaris (C; p<0.0001) when compared to the mFc control offspring. Increased volumes were seen at the lateral globus pallidus (D; p = 0.01) and neocortex (E; p = 0.02). The overall ameliorative effect of pravastatin exposure in utero is seen. Not shown are brain regions with similar volumes between groups.

Male sFlt-1 offspring showed decreased fimbria (p = 0.04), periaquaductal gray (p<0.0001), and stria medullaris (p<0.0001) volume, and increased lateral globus pallidus (p = 0.01) and neocortex (p = 0.02) volume compared with male offspring from the mFc control group (Figure 1). Prenatal pravastatin treatment restored volumes to those similar in controls (p<0.05), though only a trend was seen at the lateral globus pallidus and neocortex. Whole brain and cerebellar volumes were similar between groups.

**Figure 2:**
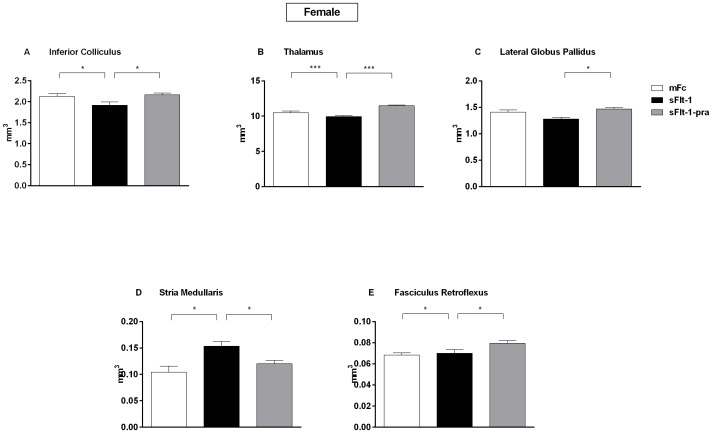
Altered Brain Volumes in 6 Month Old Female Offspring Brain. Regions of interest were analyzed by 1-way ANOVA in 6 month old female mice offspring born to pregnant CD1 mice injected either with an adenovirus carrying sFlt-1 or adenovirus carrying mFc fragment. Mice injected with sFlt-1 were treated with either pravastatin or water to result in three groups of offspring animals: mFc (n = 6), sFlt-1 (n = 3), and sFlt-1-pra (n = 10). Data are reported as mean ± SEM. The sFlt-1 group offspring showed decreased volumes at the inferior colliculus (A; p = 0.04), thalamus (B; p<0.0001), and lateral globus pallidus (C; p = 0.02) when compared to the mFc control offspring. Increased volumes were seen at the stria medullaris (D; p = 0.01), and fasciculus retroflexus (E; p = 0.01). The overall ameliorative effect of pravastatin exposure in utero is seen. Not shown are brain regions with similar volumes between groups.

Female sFlt-1 offspring showed decreased volume in the inferior colliculus (p = 0.04) and thalamus (p<0.0001) compared with female mFc control offspring (Figure 2). A similar trend was seen in the lateral globus pallidus, but only the sFlt-1-pra group reached significance when compared to sFlt-1 offspring (p = 0.02). Female sFlt-1 offspring also showed increased brain volumes at the stria medullaris (p = 0.01) and fasciculus retroflexus (p = 0.01) compared with female mFc control offspring (Figure 2). Prenatal pravastatin treatment restored volumes in all of these areas to levels similar to those seen in controls except in the fasciculus retroflexus where its effect enhanced that of preeclampsia exposure. Similar to male offspring, there were no differences in cerebellar and whole brain volumes between groups.

**Figure 3:**
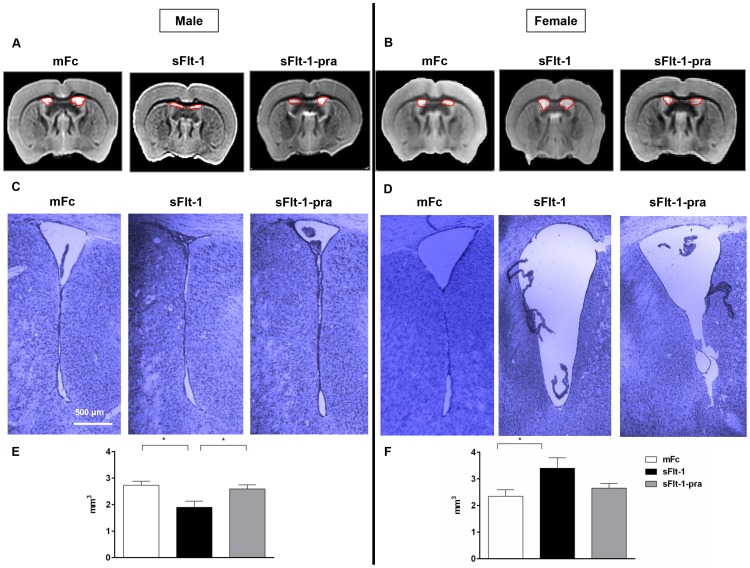
Magnetic Resonance Images of 6 Month Old Offspring Brains. Three-dimensional T2-weighted images of whole brain were obtained using a vertical bore 11.7 Tesla MRI scanner. Here in these representative coronal slices, the ventricles and periventricular tissue of male (A) and female (B) offspring are outlined in coronal view, allowing comparison between treatment groups. A decrease in ventricular volume was seen in male sFlt-1 offspring (A: sFlt-1) when compared to controls (A: mFc). Pravastatin exposure (A: sFlt-1-pra) reversed this effect. An opposite trend was seen in the female sFlt-1 offspring brain (B: sFlt-1) when compared to control (B: mFc) and pravastatin-treatment offspring (B: sFlt-1-pra). Nissl staining of coronal sections at a single ventricle and its periventricular tissue in adult offspring brains at 40 X magnification is shown in panels C and D. In these representative images, male offspring showed a decrease in volume in sFlt-1 (C: sFlt-1) offspring when compared to control (C: mFc). In-utero pravastatin exposed male offspring (C: sFlt-1-pra) showed normalization of ventricular shape and size. An increase in ventricular volume is seen in female sFlt-1 offspring brains (D: sFlt-1) when compared to control (D: mFc). Though complete resolution is not seen, there is improvement in ventricle size in the pravastatin-exposed female offspring (D: sFlt-1-pra). These representative images correlate with MRI volumetric data for male (E: mFc (n = 9), sFlt-1 (n = 3), sFlt-1-pra (n = 6)) and female (F: mFc (n = 6), sFlt-1 (n = 3), sFlt-1-pra (n = 10)) offspring brains as analyzed by 1-way ANOVA. The male sFlt-1 offspring had significantly decreased volume when compared to mFc controls and the pravastatin treatment group in males (E: p = 0.02), whereas the opposite effect was seen in female offspring (F: p<0.05).

**Figure 4:**
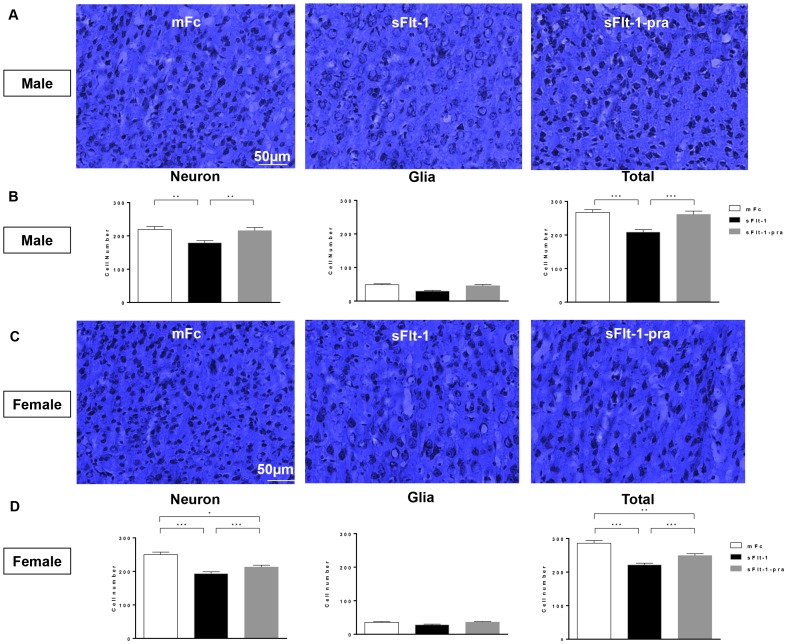
Altered Cortical Cell Counts in 6 Month Old Offspring Brain. Nissl staining of neocortex of 6 month old mice offspring shown here at 40 X magnification in panels A (male) and C (female). Neurons were identified by dark-blue positive staining. Glial cells were smaller than neurons and had darker nuclei but no clearly visible Nissl bodies in the cytoplasm. A decrease in positive staining is seen male and female sFlt-1 offspring (A: sFlt-1, C: sFlt-1) when compared to controls (A: mFc, C: mFc) and pravastatin treatment group offspring (A: sFlt-1-pra, C: sFlt-1-pra). Total cell count as well as neuronal and glial cell counts were performed on randomly chosen 7-8 fields in the frontal cortex per animal and analyzed by 2-way ANOVA (n = 3 for all groups by gender). Data are reported as mean ± SEM. Male data are seen in Panel B, and female data in Panel D. Significant decreases in total cell count are seen in both male and female sFlt-1 offspring when compared to controls (p<0.0001) and sFlt-1-pra (male and female p<0.0001), though the female sFlt-1-pra group remained significantly different from mFc controls (p = 0.001). There is no difference in glial cell count between all three groups. Neuronal cell count appears to drive this total cell count difference. The male sFlt-1 offspring (B) show a significant decrease in neurons when compared to mFc controls (p = 0.0003) and sFlt-1-pra offspring (p = 0.001). Female sFlt-1 offspring (D) show a similar drop in neuronal count versus mFc controls and sFlt-1-pra offspring (p<0.0001), with pravastatin-exposed offspring remain significantly different from mFc controls (p = 0.03).

As seen in MRI volumetric analysis, male and female offspring had opposite effects at the ventricles (Figure 3). Nissl staining correlated with these findings. The ventricles and periventricular tissue show the respective decreased and increased ventricular volume seen in male (p = 0.02) and female (p<0.05) sFlt-1 offspring when compared to mFc control offspring. The pravastatin-treatment group offspring neuroanatomy appears similar to controls, though this does not reach significance in females. On Nissl staining at the level of the neocortex, glial cells were smaller than neurons and had darker nuclei but no clearly visible Nissl bodies in the cytoplasm (Figure 4). There were no differences in neuronal size between groups. Total cell counts between groups showed a significant decrease in sFlt-1 offspring when compared to both mFc control and sFlt-1-pra offspring. This was seen in both male and female offspring brains. When analyzed by cell type, this decrease in cell number in the sFlt-1 offspring is driven only by neurons while no change was seen in glia. Moreover, the effect of pravastatin was greater in male offspring.

## Discussion

The regions of interest affected in this study participate in multiple tasks including learning, adaptation, memory, emotion, motor function, and the processing of reward, sensory input, and pain (Table 1). Those which demonstrated significant changes between treatment groups are described in Table 2. We have previously reported that adult offspring of “preeclamptic” mice display impaired performance on neuromotor testing of vestibular function, balance, and locomotor coordination in gender-specific patterns [8]. Additionally, expression of genes involved in neuronal migration, myelination, apoptosis and oxidative stress in the hypothalamus and frontal cortex were altered in these offspring (unpublished data). The correlation between prenatal insults, neurodevelopmental impairment, and neuroimaging irregularity is well established [20], [22]–[24]. Similar to our data, a mouse model of intrauterine inflammation, also a feature of preeclampsia, resulted in sex-specific long-term sequelae in offspring as measured by neurobehavioral assays, MR brain imaging, and cytochemical analysis [25]. However, our current study is the first to assess by neuro-radiology the sex-specific neuroprogramming responsible for changes in locomotor performance following exposure to a “preeclamptic” milieu, as well as the utility of maternal pravastatin prenatal treatment in preventing this fetal programming. Our neuroimaging findings are reinforced by abnormal Nissl staining which revealed decreased neuronal cell number in sFlt-1 offspring. Given our prior data showing altered gene expression of a neuron-specific migration protein, Reelin (unpublished data), this may suggest that an in-utero insult involving neuronal migration leads to the long-term anatomic and neuromotor outcomes reported. Others have also shown an association between impaired expression of Reelin at various stages of neurodevelopment and long-term adverse outcomes [23]-[24], [26]. As this protein is critical to the ultimate cellular architecture of the brain, it may offer a possible mechanism by which developmental insults translate into long-term adverse outcomes.

**Table 2:** Functions of Brain Regions with Significant Volume Changes Seen in MRI Imaging of Adult Mouse Brains.

Regions of Interest	Function
Fasciculus Retroflexus	Regulation of movement; processing of reward and motivation
Fimbria	Spatial reasoning and memory
Inferior Colliculus	Processing of auditory data
Lateral Globus Pallidus	Inhibitory regulation of voluntary movement
Neocortex	Generation of motor function, spatial reasoning, sensory perception
Periaqueductal Gray	Descending pain modulation
Stria Medullaris	Processing of pain and reward
Thalamus	Subcortical regulation of sensory and motor function
Ventricles	Storage of cerebrospinal fluid, enlarged in certain disease processes

Used in this work was an animal model of preeclampsia induced by angiogenic imbalance. This can also affect other organs such as the liver and kidneys, lead to intrauterine hypoxia, oxidative stress, and other stressors during development but resolves following delivery. This is in contrast to chronic hypertension, which is not characterized by angiogenic imbalance. These mice are not hypertensive prior to sFlt-1 overexpression nor do they remain so following delivery. The mechanism of angiogenic imbalance, overexpression of sFlt-1, also resolves following delivery [27]. Thereby this study can better mimic the transient gestational occurrence of this disease.

The association between preeclampsia and adverse neurologic outcomes and other chronic adult diseases has been well documented [9]–[14], [28]–[29]. It is thought that hypoxia, oxidative stress, and abnormal vascular and endothelial activity that occur in the setting of preeclampsia [2] contribute to an adverse uterine environment which is associated with alterations in gene expression during critical periods of fetal development resulting in long-term adverse effects in adult offspring. Statins are known to have pleiotropic properties, including improvement of endothelial dysfunction, inhibition of inflammatory and coagulation cascades, increased nitric oxide synthesis, antioxidant properties, and immunomodulatory actions [30]-[32]. It is plausible that statins improve the uterine environment during development which may ultimately prevent the fetal programming of adverse outcomes. Consistent with this, prenatal pravastatin exposure has been shown to prevent the development of adverse cardiovascular profiles in offspring of dams exposed to high cholesterol diet [33] and improve maternal vascular phenotype in our sFlt-1 model [15]–[16]. Statins also show promise in ameliorating other neurologic conditions such as hypoxic-ischemic and traumatic brain injury, Alzheimer's and Multiple Sclerosis disease models [34]–[38].

In this model, a replication-deficient adenovirus is used, and circulating levels have been documented to fall rapidly after 3 weeks [39]. Given the 2 week gestational period of CD-1 mice, if transplacental transfer is assumed, levels should fall well before weaning. This may leave a window of fetal susceptibility to transgenic overexpression. However, the designers of this vector believe that transplacental transfer is unlikely (personal communication). Furthermore, CD-1 heterozygosity for VEGF is uniformly lethal which suggests that we would have seen increased pup resorption or death in our sFlt-1 group. This did not occur, arguing against transplacental transfer of the adenovirus-sFlt-1 complex and subsequent fetal hepatic transduction [6], [39]. This is also indirectly supported by studies attempting fetal gene therapy using various adenoviruses in which only intra-amniotic or direct fetal administration of the virus vector is used to achieve adequate fetal expression [40]–[43]. Similarly, pravastatin is a hydrophilic statin and subject to efflux transporters at the placenta and blood brain barrier [44]–[48]. We therefore believe that the ability of pravastatin to achieve the changes seen in offspring born to preeclamptic dams are not due to direct effects of pravastatin on the offspring but rather through amelioration of the intrauterine environment.

In accordance with data of offspring born to preeclamptic women which showed male-dominated adverse outcomes [11]–[13], in this study, only adult male sFlt-1 offspring showed decreased volume at the fimbria and periaquaductal grey, which function in spatial reasoning, memory, and pain modulation respectively [49]–[50]. At the inferior colliculus, thalamus, fasciculus retroflexus, and lateral globus pallidus, centers of auditory and motor regulation, only females showed altered volumes [51]–[54]. Moreover, in regions where both sexes are affected, the changes in volume in adult male and female offspring were in opposite directions. Whereas males showed loss of volume at the stria medullaris, and gain in volume at the lateral globus pallidus which are involved pain, reward, and voluntary movement regulation respectively [55]–[56], females showed the opposite effect. Furthermore, the ventricular volume was decreased in males but increased in females. Advanced age, trauma, and a number of neurologic diseases are associated with increased ventricular volumes, presumably due to atrophy of surrounding parenchyma [57]–[59]. However the decrease in ventricular volume along with the neocortical volume increase seen in males may reflect pathologic cellular disorganization. This may result from altered neuronal migration or a lack of synaptic “pruning” which is essential for maturation of substructural brain connectivity and ultimate neurodevelopmental performance [60]–[62].

It is important to note, that at baseline, brain development diverges between genders. For example, diffusion tensor imaging via MRI of developing human brains has been used to study white matter architecture and underlying tracts, and in this way an overall “connectome” of the brain has been studied. Ingalhalikar, et al studied healthy human brains at ages 8–22 and correlation with brain function suggested superior motor and spatial ability in the male connectome and better memory and social cognition in the female connectome [63].

In addition to intrinsically differing pathways of brain development, when an insult occurs during the fetal period, susceptibility for long-term adverse sensorimotor and neurodevelopmental outcomes is also varies by gender. Generally, male offspring bear a greater burden of diagnoses such as cerebral palsy, autism, ADHD, and neurodevelopmental delay than their female counterparts [64]–[66]. One mechanism may be disparate circulating androgen concentrations between males and females during embryonic development which have effects on downstream brain morphology and behavior [66]. Testosterone and its metabolite dihydrotestosterone are inversely proportional to adult brain injury, whereas estrogen appears to be neuroprotective [66]. Similarly, in models of neonatal hypoxia-ischemia, male rats show increased tissue damage and behavioral effects. Infante, et al showed that adverse locomotor outcomes were seen in offspring of affected pups, and gender bias was noted, suggesting an epigenetic mechanism as well [67]. The presence of a fragile Y chromosome which is susceptible to DNA modifications such as methylation may explain this [66], [68]. Additionally, imprinting occurs at different stages based on gender, so timing of a developmental insult is also significant [69]. Other factors such as maternal diet and social interaction have been implicated in epigenetic alterations [70]. Finally, a portion of differing vulnerability to perinatal insult may be due to sex-specific cell-death pathways activated by hypoxia or oxidative stress [71]–[73].

Sex-specificity is not limited to the effect of in-utero preeclampsia exposure. Response to the pravastatin preventive therapy in this model also varies by gender. This was seen in volume restoration as measured by MRI (Figures 1–2) as well as in restoration of control-level neuronal cell counts based on Nissl staining (Figure 4). In the latter, male offspring exhibit a more significant increase in neurons in sFlt-1-pra offspring. Explanations could include a small sample size or a different dose-response to this drug in female mice. Statins have shown sex-specificity in outcomes following statin use for stroke and heart attack prevention [74]–[75]. Related study in this preeclampsia model has shown varying responses to pravastatin for both cardiovascular and metabolic parameters in offspring [76]–[77]. Future research should build on this growing literature of sex-specific effects and targeted therapy.

Our study has limitations. First, our results were obtained in an animal model of preeclampsia that is based on the angiogenic imbalances early in pregnancy. Most animal models lack some features of preeclampsia in humans thus making extrapolation or application of our results somewhat challenging clinically. Second, the lack of mFc control group treated with the pravastatin in the current work is acknowledged. The authors' aim is to consider the clinical relevance of the potential for pravastatin as a preventive therapy for women at risk for or with a history of preeclampsia. In clinical practice, there is no current circumstance in which a non-hypertensive gravida would be treated with pravastatin. Though it may be of academic interest, it would be poorly relevant to clinical practice to test the role of this drug in a control group. Given the previously reported data which showed no difference between mFc control mice treated with water and the mFc control mice treated with pravastatin, we made a decision to reserve our animal resources for treatment groups most applicable to our human patient population. Additionally, the safety of this drug in human literature has been reported in multiple studies with no evidence of teratogenicity identified [78]–[84].

Third, small sample size of sFlt-1group animals may confound our findings of statistically significant outcomes. However, this is balanced by consistency across this model in phenotypic and mRNA expression patterns (unpublished data) [8] as well as support of existing epidemiologic data [9]–[14]. Fourth, in this work, we examined offspring brains at a single time-point of 6 months, which was designed to evaluate long-term adulthood outcomes. It is possible that the findings may change with age as brain plasticity has been shown to overcome some developmental insult. Indeed, there is variation in the ages of developmental abnormalities reported from early to late childhood and adulthood in human studies [9]–[14], so further investigation at multiple time points is warranted. Finally, preeclampsia onset and severity in humans are highly variable and difficult to predict. Determining when to start pravastatin and which patients are the best candidates is likely to be challenging. Further studies may shed some light on these issues. Nevertheless, our data support human epidemiologic data by showing adverse neurologic outcomes following in utero exposure to preeclampsia.

## Conclusion

In summary, we demonstrated that adult offspring born to preeclamptic dams exhibit altered brain development which is prevented by prenatal pravastatin treatment. These data support our prior findings of abnormal neuromotor function and gene expression in this sFlt-1 mouse model of preeclampsia, the first to our knowledge to examine long-term adverse neurologic outcomes and pravastatin for prenatal prevention. This research builds on prior literature and also highlights the importance of understanding the sex-specific effects of developmental insults both in-utero and beyond as well as sex-specific response to treatment. These and other data support a role for further research into the effect of preeclampsia on long-term offspring outcomes and pravastatin as a therapy for preeclampsia prevention.
